# A *Bursaphelenchus xylophilus* effector BxICD1 inducing plant cell death, concurrently contributes to nematode virulence and migration

**DOI:** 10.3389/fpls.2024.1357141

**Published:** 2024-02-28

**Authors:** Zhiwen Li, Honghong Wang, Yuqing Cao, Xiaoling Shan, Xiaoxian He, Qiuling Huang, Kan Zhuo, Jinling Liao, Borong Lin

**Affiliations:** ^1^ College of Plant Protection, South China Agricultural University, Guangzhou, China; ^2^ Collaborative Innovation Center of Plant Pest Management and Bioenvironmental Health Application Technology, Guangdong Eco-Engineering Polytechnic, Guangzhou, China; ^3^ Guangdong Province Key Laboratory of Microbial Signals and Disease Control, South China Agricultural University, Guangzhou, China

**Keywords:** *Bursaphelenchus xylophilus*, transcriptome, *Pinus massoniana*, effector, plant cell death activation

## Abstract

The migratory endoparasitic phytonematodes *Bursaphelenchus xylophilus* is the causal agent of pine wilt disease and causes significant economic damage to pine forests in China. Effectors play a key role in the successful parasitism of plants by phytonematodes. In this study, 210 genes obtained by transcriptomics analyses were found to be upregulated in *B. xylophilus* infecting *Pinus massoniana* that were not functionally annotated nor reported previously in *B. xylophilus* infecting *P. thunbergii.* Among these differentially expressed genes, a novel effector, BxICD1, that could induce cell death in the extracellular space of *Nicotiana benthamiana* was identified. *BxICD1* was upregulated in the early stages of infection, as shown by RT-qPCR analyses. *In situ* hybridization analysis showed that *BxICD1* was expressed in the esophageal gland of nematodes. The yeast signal sequence trap system indicated that BxICD1 possessed an N-terminal signal peptide with secretion functionality. Using an *Agrobacterium*-mediated transient expression system, it was demonstrated that the cell death-inducing activity of BxICD1 was dependent on *N. benthamiana* brassinosteroid-insensitive 1-associated kinase 1 (NbBAK1). Finally, BxICD1 contributed to *B. xylophilus* virulence and migration in host pine trees, as demonstrated by RNAi silencing assays. These findings indicate that BxICD1 both induces plant cell death and also contributes to nematode virulence and migration in *P. massonian*.

## 1 Introduction

The pine wood nematode (PWN), *Bursaphelenchus xylophilus*, is a migratory endoparasitic nematode. The nematodes enter the trees through the wounds formed by *Monochamus* beetles feeding (Edwards and Linit, 1992), then colonize the tree’s xylem and cortex resin canals, obstructing water and nutrient flow. This ultimately leads to tree wilting and death (Futai, 2013), termed pine wilt disease (PWD). PWD has significant economic and ecological impacts on pine forests in Asia and Europe (Kikuchi et al., 2011). Currently, the methods for preventing and controlling PWD primarily involve directly cutting and incinerating infested trees, fumigating logs with pesticides, applying pesticides to manage *Monochamus* beetles, or injecting nematicides against *B. xylophilus* (Cui et al., 2014). However, these strategies possess certain drawbacks, including environmental pollution and significant costs. To formulate novel and eco-friendly strategies for PWD management, it is necessary to understand the mechanisms of PWN parasitism.

When plant-parasitic nematodes (PPNs) infect host plants, they secrete numerous proteins into the host. These proteins, termed effectors, play crucial roles in successful nematode parasitism, and understanding their functions is crucial to uncovering the molecular mechanisms underlying parasitism (Haegeman et al., 2012; Vieira and Gleason, 2019). Many previous studies have shown that PPN effectors mainly function in plant cell wall degradation, feeding site formation, and the suppression of host immunity (Popeijus et al., 2000; Kikuchi et al., 2006; Kudla et al., 2007; Vanholme et al., 2007; Chen et al., 2017; Verma et al., 2018; Chen et al., 2021; Song et al., 2021). Most studies have focused on sedentary endoparasitic nematodes, such as root-knot nematodes and cyst nematodes. Recently, some effectors from *B. xylophilus* have been characterized, and effectors with plant cell wall-degradation or host immunity suppression functions have been discovered (Kikuchi et al., 2006; Wen et al., 2021, Wen et al., 2022; Hu et al., 2022a; Qiu et al., 2023). Intriguingly, a number of effectors from *B. xylophilus* were found to induce host cell death. Of these, BxCDP1 can trigger plant cell death (PCD) to induce *Pinus thunbergii* resistance against nematodes (Hu et al., 2020; Hu et al., 2022b). Several other effectors, such as BxSapB1, BxSapB2, and BxSapB3, while capable of inducing plant cell death, also function as a virulence factor, during *B. xylophilus* infection (Huang et al., 2019; Hu et al., 2019; Zhao et al., 2020). This phenomenon is relatively common in effectors from fungi and oomycetes, such as the *Fusarium graminearum* effector Fg62 (Wang et al., 2023), the *Phytophthora infestans* effector Pi23226 (Lee et al., 2023), etc., which can induce host cell death and promote pathogen parasitism.

Masson pine (*Pinus massoniana*) is a pioneer species for afforestation timber and oleoresin production, widely distributed in South China. However, it is highly sensitive to *B. xylophilus* (Xue et al., 2013; Liu et al., 2022a). Despite this, the roles of effectors during *B. xylophilus* parasitism in *P. massoniana* are poorly understood. In this study, transcriptomes of *B. xylophilus* feeding on *Pestalotiopsis* sp. and infecting *P. massoniana* were analyzed by RNA sequencing (RNA-seq). Differentially expressed genes of *B. xylophilus* were identified in these two conditions, which were analyzed to identify candidate effectors involved in *B. xylophilus* parasitism in *P. massoniana* parasitism. Among the candidate effectors, a novel effector (named BxICD1) that was expressed in the esophageal gland of *B. xylophilus* and upregulated during nematode parasitism in *P. massoniana* was identified. Here, we showed that the effector BxICD1 not only induces PCD, but also promotes nematode virulence and migration in *P. massoniana*.

## 2 Materials and methods

### 2.1 Nematode and plant materials

*B. xylophilus* was isolated from a wilted Masson pine in Guangdong province, China, purified using a single fertilized adult female, and cultured in the fungus *Pestalotiopsis* sp. grown on potato dextrose agar (PDA) medium at 25°C under 16 h light/8 h dark (16/8 LD) conditions for 6 days.

Approximately 3-year-old *P. massoniana* seedlings were obtained from Yangshan County (24°17′1.6″N, 112°33′42.4″E), Qingyuan City, Guangdong province, China, and grown at temperatures ranging from 25°C to 30°C. *Nicotiana benthamiana* LAB were grown in a glasshouse at 25°C with a relative humidity of 60% under 16/8 LD conditions.

### 2.2 RNA extraction and cDNA synthesis

*B. xylophilus* was inoculated into a small, approximately 1 cm, wound in *P. massoniana* stems. After 12 h, mixed-life-stage nematodes were isolated from the pine stems using a Baermann funnel technique (Hu et al., 2019), and approximately 20,000 nematodes were used for RNA extraction. The same number of *B. xylophilus* nematodes were also isolated from *Pestalotiopsis* sp. grown on PDA plates for RNA extraction. Each treatment consisted of two replicates. Total RNA was extracted using the RNAprep Pure Micro Kit (DP420, TianGen Biotech), and first-strand cDNA was synthesized from 1 µg of total RNA using a TransScript One-Step gDNA Removal and the cDNA Synthesis SuperMix kits (AT311, TransGen Biotech, Beijing, China) according to the manufacturer’s instruction.

### 2.3 RNA-seq analysis

Sequencing libraries were constructed using the NEBNext Ultra RNA Library Prep Kit (E7530, NEB, USA) according to the manufacturer’s instructions. RNA integrity was determined by the RNA Nano 6000 Assay Kit of the Bioanalyzer 5400 system (Agilent Technologies, CA, USA). Total RNA was used as input material for the RNA library preparations. The library preparations were sequenced on an Illumina Novaseq platform, generating 6.49-6.82 GB of data of 150-base long paired reads per sample (Modi et al., 2021). The raw RNA-Seq data in this study are available through the National Center for Biotechnology Information under the accession number PRJNA1027064.

Paired-end reads were aligned to the reference genome (https://parasite.wormbase.org/Bursaphelenchus_xylophilus_prjea64437/Info/Index) using Hisat2 v2.0.5 (Kim et al., 2019). The reads mapped to each gene were measured using FeatureCounts v1.5.0-p3 (Liao et al., 2014). The FPKM values of each gene were calculated based on the length of the gene and the reads count mapped to this gene (Trapnell et al., 2010).

Differential expression analysis was performed using the DESeq2 R package (1.20.0). The resulting *P*-values were adjusted using the Benjamini and Hochberg’s approach for controlling the false discovery rate (Benjamini and Hochberg, 1995). Genes identified by DESeq with an adjusted *P*-value<0.05 and |Log_2_(fold change)|>1 were considered differentially expressed genes.

### 2.4 Real-time quantitative PCR assays and data analysis

Real-time quantitative PCR (RT-qPCR) was performed using Green qPCR SuperMix (AQ101, TransGen Biotech, Beijing, China) on a Thermal Cycler Dice Real Time System (Takara, Beijing, China). The RT-qPCR reaction mixture consisted of 1.0 μL cDNA, 0.4 μL of each of the 10 pM primers, 10 μL 2 × Green qPCR SuperMix, and 8.2 μL RNase-free ddH_2_O. The ubiquitin-conjugating enzyme gene *BxUBI2* (GenBank accession number CAD5208953.1) and *NbEF1*α (Heese et al., 2007) were used as endogenous controls for gene expression normalization in the different species. All the gene-specific primers used in this study are listed in **Supplementary Table 1**. The RT-qPCR cycling conditions were as follows: 94°C for 30 sec, followed by 40 cycles of 94°C for 10 s, 55°C annealing for 15 s, 72°C elongation for 10 s. The relative changes in gene expression were determined using the 2^−ΔΔCT^ method (Livak and Schmittgen, 2001). Three independent experiments were conducted.

### 2.5 Screening of putative secreted proteins

Putative secreted proteins encoded by differentially expressed genes (DEGs) were predicted using both SignalP 5.0 (https://services.healthtech.dtu.dk/service.-php?SignalP-5.0) and TMHMM-2.0 (https://services.healthtech.dtu.dk/service.php?TMHMM-2.0) for the presence of an N-terminal signal peptide (SP) and for the absence of a transmembrane domain, respectively. These steps were implemented as a ‘secreted protein prediction’ workflow (Petitot et al., 2016). Protein functional domains were predicted by EGGNOG-MAPPER (http://eggnog-mapper.embl.de/). The phylogenetic tree was constructed using the NJ method and visualized using MEGA-X (Kumar et al., 2018).

### 2.6 Gene amplification

The full-length cDNA sequences of 10 candidate genes were amplified by using the corresponding gene-specific primers. The PCR reaction was performed in a total volume of 50 μL containing 1.0 U KOD-FX (KFX-101, Toyobo, Osaka, Japan), 25 μL 2 × PCR Buffer, 10 μL 2 mM dNTPs, 1.5 μL of each of the 10 pM primers, 200 ng cDNA, and sterilized distilled water up to 50 μL. The PCR cycling conditions were as follows: 95°C for 5 min, followed by 30 cycles of 98°C for 10 s, annealing at 55°C for 30 s, elongation at 68°C for 30 s, and final elongation at 68°C for 5 min.

### 2.7 Plasmid construction

The CaMV35S driven *β-glucuronidase* (*GUS*) gene of the pCAMBIA1305.1 vector was removed and replaced with the *eGFP* gene to generate the binary vector pCambia1305.1^ΔGUS^ and pCambia1305.1:eGFP. To construct the overexpression vector, the 10 candidate genes mentioned above, with or without the native SP sequence, were amplified and ligated to the linearized pCambia1305.1:eGFP digested by *Nco*I and *Pml*I (FD0574 and FD0364, Thermo Fisher Scientific, MA, USA) to generate pCambia1305.1:gene:eGFP, following the instructions of the ClonExpress II One Step Cloning Kit (C112-01, Vazyme, Nanjing, China). *BxICD1*:*Flag* was amplified and ligated to the linearized pCambia1305.1^ΔGUS^ to generate pCambia1305.1:BxICD1:Flag. For pSUC2, vectors were digested by *Eco*RI and *Xho*I (FD0274 and FD0694, Thermo Fisher Scientific, MA, USA) in the appropriate conditions. The SP sequences of *BxICD1* and *Avr1b* were linked to linearized pSUC2 to generate pSUC2:SP^BxICD1^ and pSUC2:SP^Avr1b^ following the instructions of the ClonExpress II One Step Cloning Kit.

### 2.8 *In situ* hybridization experiments

Approximately 10,000 mixed-life-stage nematodes were collected from pine stems. The primer pairs ISH-BxICD1-F/ISH-BxICD1-R were employed to synthesize digoxygenin (DIG)-labeled sense and antisense DNA probes based on the *BxICD1* fragments of 96–281 bp using a PCR DIG Probe Synthesis Kit (1636090910, Roche Applied Science, Rotkreuz, Switzerland). The primer pair ISH-BxICD2-F/ISH-BxICD2-R was employed to synthesize sense and antisense DNA probes based on the *BxICD2* fragments of 103-311 bp in size. The *in situ* hybridization and staining of the nematodes was performed as described previously (De Boer et al., 1998). Nematodes were then examined using a Nikon ECLIPSE Ni microscope (Nikon, Tokyo, Japan).

### 2.9 Cell death assay

The cell death assay was performed as described previously (Chen et al., 2017). Briefly, the pCambia1305.1:gene:eGFP constructs mentioned above were individually introduced into *Agrobacterium tumefaciens* GV3101. Then the transformed bacteria were suspended in a buffer containing 10 mM 2-(N-morpholino) ethanesulfonic acid (MES) (pH 5.5) and 200 μM acetosyringone until a 600 nm absorbance (OD_600_) of 0.6 was reached. Then, the *A. tumefaciens* cell suspensions were infiltrated into 4-week-old *N. benthamiana* leaves. After 3 days, the cell-death phenotype was observed and photographed. *eGFP* and the *P. infestans* elicitor gene *INF1*, cloned individually into the pCambia1305.1 vector, were used as the negative and positive control, respectively. The infiltration assay was performed three times, and five different plants with two inoculated leaves were used in each assay.

### 2.10 Yeast signal sequence trap system

The secretion function of the *BxICD1* SP was verified using the yeast signal sequence trap system described previously (Yin et al., 2018). Briefly, the SP sequence of *BxICD1* was cloned into the pSUC2 vector containing the invertase gene but lacking Methionine (Met) and SP sequence to generate pSUC2:SP^BxICD1^-Invertase. Additionally, the SP of *Phytophthora sojae* RXLR effector Avr1b has been demonstrated to have secretion function in yeast (Dou et al., 2008) and was also cloned into pSUC2 to generate pSUC2:SP^Avr1b^-Invertase as a positive control. pSUC2:SP^BxICD1^-Invertase and pSUC2:SP^Avr1b^-Invertase were transformed into the yeast strain YTK12 by the lithium acetate method (Gietz and Schiestl, 2007; Yin et al., 2018). The yeast strains YTK12 and YTK12 carrying the pSUC2 empty vector were used as negative controls. YTK12 and YTK12 containing pSUC2-derived plasmids were grown on a CMD-W medium (0.67% yeast N base without amino acids, 0.075% W dropout supplement, 2% sucrose, 0.1% glucose, and 2% agar) to detect the expression of pSUC2-derived plasmids. The enzymatic activity of invertase was confirmed by the reduction of the dye 2,3,5-triphenyltetrazolium chloride (TTC) to the insoluble red-colored 1,3,5-triphenylformazan (TPF).

### 2.11 Subcellular localization

The subcellular localization assays were performed as described previously (Chen et al., 2017). Briefly, *BxICD1* sequences with or without the native SP were cloned into the pCambia1305.1 vector to generate BxICD1:eGFP and BxICD1^Δsp^:eGFP, respectively. *eGFP* alone was used as the control. The constructs were used for the transformation of 4-week-old *N. benthamiana* leaves by agroinfiltration. Then the *N. benthamiana* plants were cultured for 3 days at 25°C under 16/8 LD. *N. benthamiana* leaves expressing the constructs were infiltrated with 30% glycerol for 20 min to induce plasmolysis for the observation of extracellular localization (Chen et al., 2021). The fluorescence was observed using an SP5 Leica confocal microscope (Nikon, Tokyo, Japan).

Western blotting was performed to verify the production of intact BxICD1:eGFP and BxICD1^Δsp^:eGFP fusion proteins in *N. benthamiana*, as described previously (Chen et al., 2021). Briefly, the total proteins from *N. benthamiana* leaves were extracted using RIPA lysis buffer. The proteins were denatured, separated on an SDS-PAGE gel, and transferred to a nitrocellulose membrane (PALL, Washington, NY, United States). After blocking with 5% (w/v) non-fat milk for 2 h at room temperature, the membranes were incubated with a primary mouse anti-GFP antibody (1:5000 dilution) (HT801, TransGen Biotech, Beijing, China) in a blocking solution for 2 h. Then membranes were incubated with an anti-mouse horseradish peroxidase-conjugated secondary antibody at a 1:5000 dilution (HS201, TransGen Biotech, Beijing, China). Proteins were visualized using the Immobilon Western Chemiluminescent system with Pierce ECL Western Blotting Substrate (Thermo Fisher Scientific, MA, USA). Ponceau S staining was used to assess equal loading (Goldman et al., 2016).

### 2.12 Electrolyte leakage assay

Electrolyte leakage assays were performed in *N. benthamiana*, as described previously (Yu et al., 2012). The *A. tumefaciens* harboring BxICD1:eGFP, BxICD1^Δsp^:eGFP, eGFP and INF1 were infiltrated into *N. benthamiana* leaves as described above. 36 h after agroinfiltration, five leaf discs (9 mm diameter) were floated on 5 mL distilled water for each sample and shaken at 25°C for 3 h. Then the conductivity of the solution was measured with a conductivity meter (DDS-307A, Shanghai INESA Scientific Instrument) to obtain the value A. In addition, the conductivity of the solution containing the leaf discs was measured after boiling for 30 min to obtain the value B. Ion leakage was calculated according to the formula (value A/value B) × 100%. The experiment was performed three times. Western blotting was performed to detect protein expression as described above.

### 2.13 In planta RNAi

Virus-induced gene silencing targeting *NbBAK1* and *NbSOBIR1* in *N. benthamiana* was performed as described previously (Lin et al., 2016). Briefly, the vectors pTRV1, pTRV2:*NbBAK1*, pTRV2:*NbSOBIR1*, and pTRV2:*eGFP* were individually transformed into *A. tumefaciens* GV3101. *A. tumefaciens* GV3101 harboring pTRV1 was mixed in a 1:1 ratio with those harboring pTRV2:NbBAK1, pTRV2:*NbSOBIR1* or pTRV2:*eGFP*, respectively, and infiltrated into *N. benthamiana* leaves. The silencing efficiency of *NbBAK1* and *NbSOBIR1* was validated by RT-qPCR. RT-qPCR was performed as described above. The infiltration experiment was performed thrice and three different plants, with three inoculated leaves in each, were used in each assay.

### 2.14 *In vitro* RNAi

The *BxICD1* gene was silenced using *in vitro* RNAi, as described previously (Liu et al., 2022b). Briefly, the T7-promoter sequence was introduced in the sense and antisense direction of a 186-bp *BxICD1* fragment using the primer pairs RNAi-T7BxICD1-F/RNAi-BxICD1-R and RNAi-BxICD1-F/RNAi-T7BxICD1-R. The PCR was performed as described above. The obtained PCR products were used to synthesize double-stranded RNA (dsRNA) using the Thermo T7 Transcription Kit (TSK-101, Toyobo, Shanghai, China) according to the manufacturer’s instructions. The same approach was used to synthesize dsRNA for *eGFP* using the primer pairs RNAi-T7eGFP-F/RNAi-eGFP-R and RNAi-eGFP-F/RNAi-T7eGFP-R. Nematodes were then inoculated in a dsRNA and a non-dsRNA solution at 25°C for 24 h. The treated nematodes were washed with ddH_2_O three times to remove external dsRNA. Subsequently, approximately 5,000 nematodes were collected for RNA extraction. The remaining nematodes were used for nematode virulence, migration, and reproduction analysis. The extent of *BxICD1* silencing was assessed using RT-qPCR. RNA extraction and RT-qPCR were performed as described above, except for the different primer pairs that were used in the RT-qPCR.

### 2.15 Infection assay

Each 3-year-old *P. massoniana* seedling was inoculated with 1,000 *B. xylophilus* treated with a *BxICD1* dsRNA, *eGFP* dsRNA, and non-dsRNA solution. Nine to ten pine trees were inoculated with each treatment. The experiment was repeated 3 times. Following the classification by Hu et al. (2022a), the morbidity degree of the *P. massoniana* seedlings was categorized into five different grades: 0, all needles were green; I, a few needles have turned yellow; II, approximately half of the needles have turned yellow or brown; III, most of the needles turned brown; and IV, the entire seedling was withered. The following formulas were used to calculate morbidity and the disease severity index.


$$\text{morbidity }\left(\%\right)=\frac{\text{Total number of diseased seedlings}}{\text{Total number of seedlings inoculated}}\times 100$$

$$\text{Disease severity index}\left(\text{DSI}\right)=\frac{\sum^{\;}\text{Total number of diseased seedlings}\times \text{morbidity degree}}{\text{Total number of seedlings}\times \text{the highest morbidity degree}}\times 100$$

### 2.16 Migration assay

The migration ability of *B. xylophilus* in *P. massoniana* was determined as described previously (Kusumoto et al., 2014). Briefly, approximately 800 nematodes treated with *BxICD1* dsRNA, *eGFP* dsRNA, and non-dsRNA solutions were used to inoculate 3-year-old *P. massoniana* seedlings. 12 h after inoculation, the pine stem segments 1-2, 2-3, 3-4 and 4-5 cm below the inoculation point were used for nematode extraction using the Baermann funnel method. Nematodes isolated from these stem segments were counted. The experiment was repeated twice with four biological replicates each time. Statistically significant differences between treatments and controls were determined by Duncan’s multiple-range test.

### 2.17 Reproduction and feeding rate analysis

The reproduction and feeding rate of *B. xylophilus* in fungi was assessed as described previously (Hu et al., 2022a). Approximately 100 nematodes treated with *BxICD1* dsRNA, *eGFP* dsRNA, and non-dsRNA solutions were inoculated to PDA plates covered with *Pestalotiopsis* sp. After culturing at 25°C for 8 d, the nematodes were collected from each PDA plate using the Baermann funnel method and counted to calculate their reproduction. The amount of *Pestalotiopsis* sp. remaining on PDA plates was measured to determine the feeding rate of *B. xylophilus*. Six PDA plates were used for each treatment, and the experiment was repeated three times. Statistically significant differences between treatments and controls were determined by Duncan’s multiple-range test.

## 3 Results

### 3.1 RNA-seq data of *B. xylophilus*

RNA was extracted from *B. xylophilus* during the mycetophagous and phytophagous stages to construct respective RNA-Seq libraries. In total, 181,144,358 raw reads were obtained from all samples. After filtering out low-quality sequences, 177,125,722 clean reads were retained. The GC content of individual libraries was between 48%-49%. On average, 92% of the clean reads mapped to the *B. xylophilus* reference genome (**Supplementary Table 2**). The high genome coverage of our RNA-Seq data indicated that the transcriptome data were reliable for the further bioinformatics analyses.

### 3.2 Identification of PCD-inducing effectors

**Figure 1:**
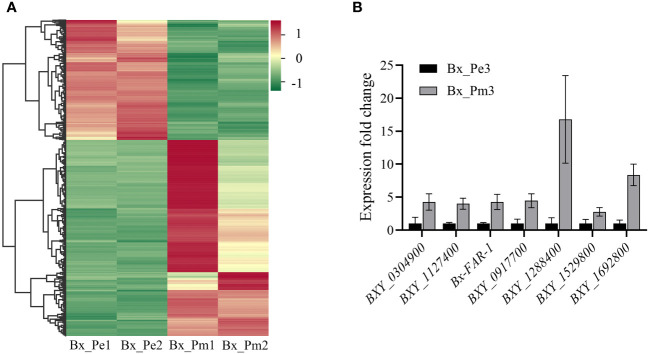
Differentially expressed genes at the mycetophagous and phytophagous stages of *Bursaphelenchus xylophilus*. **(A)** Heatmap of differentially expressed genes at the mycetophagous and phytophagous stages. **(B)** Validation of differentially expressed genes (DEGs). Validation of seven DEGs identified in the RNA-seq dataset by quantitative reverse transcription PCR. The relative expression levels were calculated using the 2^−ΔΔCt^ method; data represent shown as the mean ± standard deviation (SD) of three replicated; the experiments were performed twice with similar results. Bx_Pe1, Bx_Pe2, and Bx_Pe3 correspond to the three *B*. *xylophilus* samples from the mycetophagous stage; Bx_Pm1, Bx_Pm2, and Bx_Pm3 correspond to the three *B*. *xylophilus* samples from the phytophagous stage.

Differential expression analysis showed that 1,233 genes were differentially expressed between the mycetophagous and phytophagous stages of *B. xylophilus*. Compared with the mycetophagous stage, 765 genes were significantly up-regulated, and 468 genes were significantly down-regulated in the phytophagous stage (**Figure 1A**). To validate the identified DEGs based on the RNA-seq data, seven genes were selected and validated by RT-qPCR. Similar expression levels were observed for all seven genes in both RNA-seq and RT-qPCR datasets, further confirming the reliability of the RNA-seq transcriptome data (**Figure 1B**).

**Figure 2:**
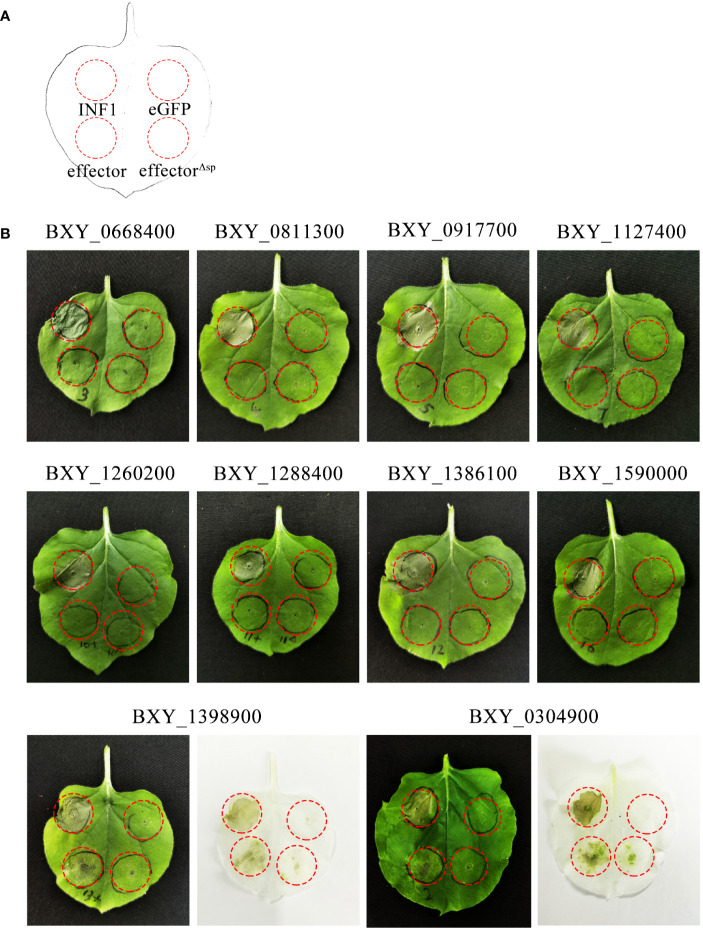
Screening of candidate effectors from *Bursaphelenchus xylophilus* capable of inducing plant cell death. **(A)** Schematic diagram of the location of *Agrobacterium tumefaciens*-injected tobacco leaves. INF1 is *Phytophthora infestans* elicitin, a known apoplastic protein that induces cell death in *Nicotiana benthamiana*, used as a positive control. eGFP, an enhanced green fluorescent protein that cannot induce cell death, was used as a negative control. “Effector” represents candidate effectors with the native signal peptide; “effector^Δsp^” represents candidate effectors without the native signal peptide. **(B)** Ten candidate effectors were expressed in *N. benthamiana* leaves. The infiltration assay was performed thrice, and five different plants with two inoculated leaves were used in each assay. Similar results were obtained from all experiments.

Among the 765 up-regulated genes, 394 encoded proteins contained an SP and lacked transmembrane domains, as predicted by SignalP 5.0 and TMHMM-2.0. Of these, 210 genes had no functional annotations and were not identified in the transcriptome analysis of *B. xylophilus* infecting *Pinus thunbergii*. 31 of these 210 genes were further considered to be candidate genes encoding for effectors (**Supplementary Table 3**) based on the following criteria: Log_2_(fold change) > 3 and FPKM > 10 with regards to their differential expression, and a gene open reading frame (ORF) is smaller than 600 bp. From this set, 10 genes were randomly selected and were transiently expressed in *N. benthamiana*. The results showed that the genes BXY_0304900 and BXY_1398900 can induce cell death (ICD) in the presence of the native SP. They were named BxICD1 and BxICD2, respectively (**Figure 2**).

**Figure 3:**
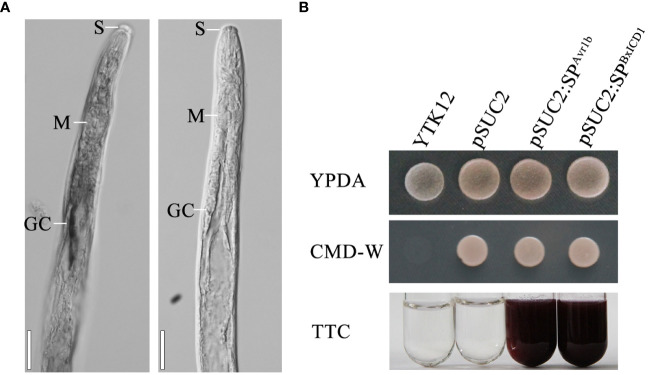
Localization of *BxICD1* mRNA and secretion function analysis of BxICD1 signal peptide. **(A)** Localization of *BxICD1* mRNA in esophageal gland cells of *Bursaphelenchus xylophilus* by *in-situ* hybridization. Fixed nematodes were hybridized with digoxigenin-labeled antisense (left) and sense (right) cDNA probes from *BxICD1*. S, stylet; M, median bulb; GC, esophageal gland. Scale bars = 50 µm **(B)** Secretion function analysis of the BxICD1 signal peptide. The predicted signal peptide of BxICD1 was cloned into the yeast vector pSUC2 to generate pSUC2:SP^BxICD1^-invertase constructs, and pSUC2: SP^Avr1b^-Invertase was used as a positive control. YTK12 can grow on YPDA plates. CMD-W media was used to ensure the expression of pSUC2-derived plasmids. Invertase can reduce 2,3,5-Triphenyltetrazolium Chloride (TTC) to insoluble red-colored 1,3,5-Triphenylformazan (TPF).

The nematode tissue localization of *BxICD1* and *BxICD2* in the nematodes was further determined by *in situ* hybridization. Hybridization signals were detected in the esophageal gland cells with the DIG-labeled antisense cDNA probe of *BxICD1* and in the intestinal terminus with the antisense cDNA probe of *BxICD2*. No hybridization signals were detected in *B. xylophilus* tissues with the sense cDNA probe of *BxICD1* and *BxICD2* (**Figure 3A**, **Supplementary Figure 1**). The results indicated that *BxICD1* should be a nematode effector because it was expressed in the typical secretory organs of nematodes. Therefore, we selected the effector BxICD1 for further analysis.

### 3.3 BxICD1 contains a SP with a secretion function

The *BxICD1* gene has a 396-bp ORF, which encodes a 131-amino acid protein. The protein possesses a 19-amino acid SP, as predicted by the SignalP program, and has no putative transmembrane domain according to TMHMM. It also shows similarities to the proteins of *B. xylophilus* by a protein BLAST search, including CAD5219553 (62.3% identity), CAD5219557 (44.6% identity), CAD5219551 (40.2% identity) etc., while no sequences were matched to other organisms. A neighbor-joining (NJ) tree was constructed to examine the phylogenetic relationships among these proteins, with *Bx*ICD1 and CAD5219553 being located in the same branch (**Supplementary Figure 2B**).

To determine whether the SP of BxICD1 is functional, the predicted SP was cloned into the yeast vector pSUC2. The growth of yeast YTK12 yeast carrying pSUC2, pSUC2:SP^BxICD1^ and pSUC2:SP^Avr1b^ constructs on CMD-W demonstrated the successful transformation with the plasmids. The yeast strain YTK12 carrying pSUC2:SP^BxICD1^-Invertase or the positive control pSUC2:SP^Avr1b^-Invertase could reduce TTC to the red-colored TPF. By contrast, no color change was observed in the YTK12 strain used as a negative control, and YTK12 carried the empty pSUC2 vector (**Figure 3B**). These results suggested that BxICD1 carries a functional secretory SP.

### 3.4 Apoplastic localization of BxICD1 is required for cell death induction in *N. benthamiana*

**Figure 4:**
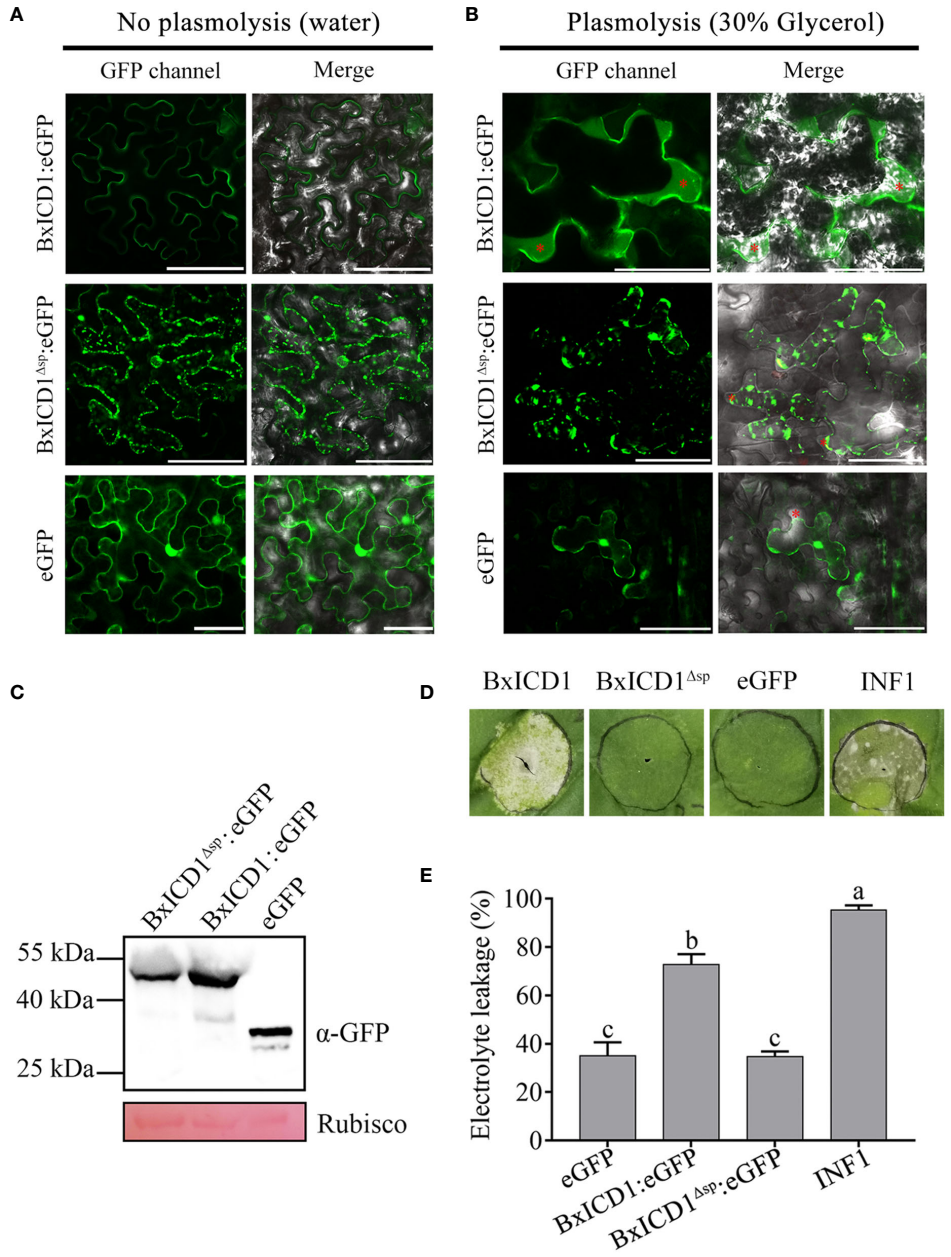
Apoplastic localization of BxICD1 is required for the induction of cell death in *Nicotiana benthamiana*. **(A)** *Agrobacterium* strain GV3101 carrying the fusion constructs BxICD1:eGFP, BxICD1^Δsp^:eGFP and the eGFP control were infiltrated in *Nicotiana benthamiana* leaves for transient expression analysis. **(B)** *N. benthamiana* leaves expressing BxICD1:eGFP, BxICD1:eGFP^Δsp^ and eGFP were treated with 30% glycerol for plasmolysis. GFP signals were observed at 3 days after infiltration. The red asterisks indicate the apoplast region. Scale bars=50 µm. **(C)** BxICD1-triggered cell death in *N. benthamiana*. *Agrobacterium* strain GV3101 carrying the constructs BxICD1:eGFP, BxICD1^Δsp^:eGFP, eGFP, and INF1 were infiltrated in *N. benthamiana* leaves for transient expression analysis. The leaf phenotypes were observed after 3 days. **(D)** Quantification of cell death by measuring electrolyte leakage in *N. benthamiana* leaves at 3 days post-infiltration with constructs encoding the indicated proteins. Data represent the mean of three repeats ± SD. Three independent experiments were performed with similar results, with three technical replicates for each reaction. **(E)** Western blot analysis was performed to confirmed the expression of proteins in *N. benthamiana*. Ponceau S staining of RuBisCO was used as indicate the protein loading control.

Based on the above results, BxICD1 could induce PCD in the presence of the native SP, therefore the subcellular localization of BxICD1 was determined. Three constructs, BxICD1:eGFP, BxICD1^Δsp^:eGFP and eGFP were generated and transiently expressed in *N. benthamiana* leaves. Based on the results, the BxICD1:eGFP fusion protein was accumulated in the edges of the tobacco cell, while BxICD1^Δsp^:eGFP and eGFP were localized in the cytoplasm (**Figure 4A**). Plasmolysis was further performed to distinguish the plasma membrane from the apoplast. After the plasmolysis treatment, BxICD1:eGFP fusion proteins were observed in the extracellular space, while BxICD1^Δsp^:eGFP and eGFP were still localized in the intracellular space (**Figure 4B**). Western blot analysis using a GFP antibody revealed bands at approximately 42, 40 and 27 kDa, corresponding to BxICD1:eGFP, BxICD1^Δsp^:eGFP, and eGFP, respectively (**Figure 4C**), indicating that BxICD1:eGFP and BxICD1^Δsp^:eGFP fusion proteins were expressed intact. At the same time, BxICD1:eGFP could induce tobacco cell death, while BxICD1^Δsp^:eGFP and eGFP could not (**Figure 4D**). In addition, the electrolyte leakage induced by INF1 or BxICD1:eGFP was significantly greater than that induced by BxICD1^Δsp^:eGFP or eGFP (**Figure 4E**). These results suggested that the presence of BxICD1 in *N. benthamiana* apoplasts triggers cell death.

### 3.5 BxICD1-triggered PCD depends on NbBAK1

**Figure 5:**
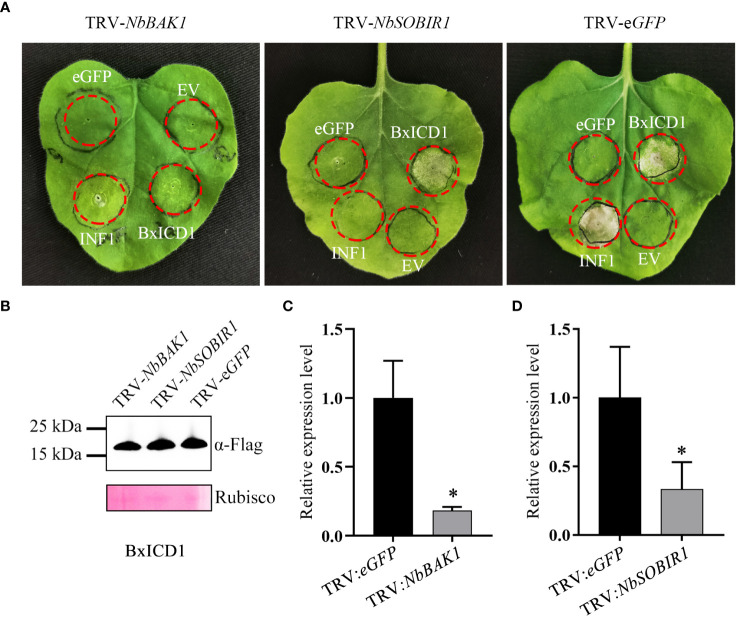
BxICD1-triggered cell death depends on NbBAK1. **(A)** NbBAK1, but not NbSOBIR1, is required for BxICD1-triggered cell death in *Nicotiana benthamiana*. *N. benthamiana* plants were subjected to VIGS by inoculation with TRV constructs (TRV:*eGFP*, TRV : *NbBAK1, or* TRV : *NbSOBIR1*). Three weeks after inoculation, BxICD1, INF1, the empty vector (EV), and eGFP were transiently expressed in the gene-silenced leaves and photographed after 3 d. The infiltration experiment was performed thrice, with three different plants with three inoculated leaves were used in each assay. All experiments were performed with similar results. **(B)** Western blot analysis confirmed the expression of BxICD1 protein in gene-silenced plants. Ponceau S staining of RuBisCO was used as indicate the protein loading control. **(C, D)** The relative expression levels of *NbBAK1* and *NbSOBIR1* in gene-silenced plant were determined by RT-qPCR. Data represent the mean of three replicates ± SD. Three independent experiments were performed with similar results. Asterisks indicate significant differences (*P*<0.05).

Many studies have demonstrated that BAK1 and SOBIR1 are indispensable for cell death triggered by many apoplastic effectors (Liebrand et al., 2014). To determine whether NbBAK1 and NbSOBIR1 are involved in BxICD1-triggered cell death, they were knocked down using virus-induced gene silencing (VIGS) in *N. benthamiana*. Two weeks after viral inoculation, *N. benthamiana* plants were agroinfiltrated with the pCAMBIA1305.1:BxICD1:Flag, pCAMBIA1305.1:INF1, and pCAMBIA1305.1:eGFP : Flag-expressing constructs, respectively. Based on the results, INF1 failed to induce cell death in *NbBAK1-* or *NbSOBIR1*-silenced plants. Notably, although BxICD1 was unable to induce cell death in *NbBAK1-*silenced plants, it retained the capacity to induce cell death in *NbSOBIR1*-silenced plants (**Figure 5A**). Neither eGFP nor the EV, used as negative controls, triggered cell death in pTRV2:*NbBAK1-*, pTRV2:*NbSOBIR1-* and pTRV2:*eGFP*- infiltrated plants (**Figure 5A**). Western blot analysis was used to confirm BxICD1 protein expression in *NbBAK1*-, *NbSOBIR1*- and *eGFP*-silenced plants (**Figure 5B**), and RT-qPCR confirmed the successful silencing of *NbBAK1* and *NbSOBIR1* in plants (**Figures 5C, D**). Taken together, these results indicated that BxICD1-triggered cell death is dependent on NbBAK1.

### 3.6 *BxICD1* affects *B. xylophilus* virulence and migration in *P. massoniana*

**Figure 6:**
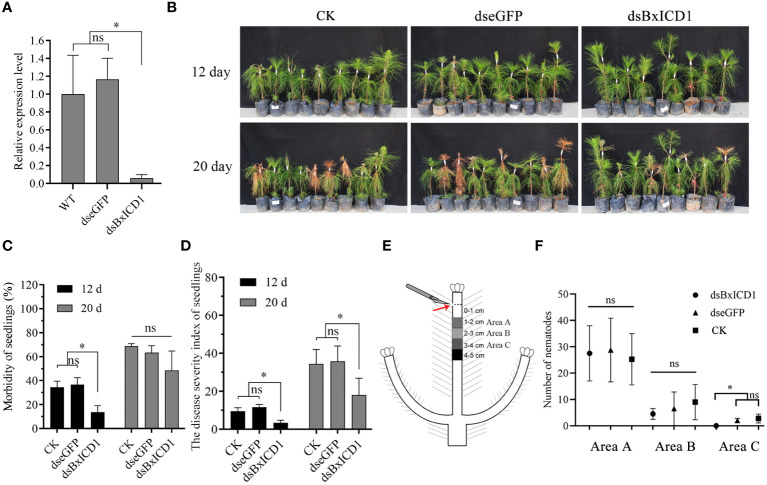
BxICD1 contributes to the virulence and migration of *Bursaphelenchus xylophilus*. **(A)** *BxICD1* silencing efficiency after treatment with *BxICD1* dsRNA in *B*. *xylophilus*. Asterisks indicate significant differences (*P*<0.05). ns indicates no significant differences (*P*≥0.05). **(B)** Representative photographs of *Pinus massoniana* seedlings at 12 days and 20 days post-inoculation. dseGFP, dsBxICD1 and CK correspond to nematodes inoculated in *eGFP*-, *BxICD1-* and non-dsRNA solution, respectively. **(C)** The morbidity of *Pinus massoniana* seedlings inoculated with dseGFP-, dsBxICD1-, and non-dsRNA solution -treated nematodes. **(D)** The disease severity index of *P. massoniana* seedlings inoculated with dseGFP-, dsBxICD1-, and non-dsRNA solution-treated nematodes. **(E)** Schematic depiction of nematode inoculation and sampling. **(F)** The number of nematodes in Area A, Area B, and Area C wood sections under three different treatments. “ns” indicates no sigificant difference (p≥0.05).

To assess the role of *BxICD1* in nematode parasitism, an RNAi assay was performed by soaking *B. xylophilus* in *BxICD1* dsRNA. The transcript levels of *BxICD1* were significantly decreased after incubation with the *BxICD1* dsRNA, compared to the treatments with eGFP dsRNA and non-dsRNA solution (**Figure 6A**), demonstrating that RNAi was successful.

Subsequently, inoculation experiments were performed using *B. xylophilus* that underwent different pre-treatment. Compared with the *eGFP* dsRNA-treated and H_2_O-treated nematodes at 12 dpi, *BxICD1* dsRNA-treated nematodes caused to a significantly lower morbidity and disease severity index (DSI) in *P. massoniana*. The morbidity was calculated at 36.7%, 34.4%, and 13.7% after inoculation with *eGFP* dsRNA-, H_2_O- and *BxICD1* dsRNA-treated nematodes, respectively (**Figure 6B**), while corresponding DSI was 11.7, 9.5, and 3.4 (**Figure 6C**). At 20 dpi, the DSI of *P. massoniana* inoculated with *BxICD1* dsRNA-treated nematodes was still significantly lower compared with *P. massoniana* inoculated with *eGFP* dsRNA and non-dsRNA solution-treated nematodes. However, no significant differences in morbidity were observed (**Figures 6C, D**). Moreover, at 12 h post-inoculation, *B. xylophilus* was isolated from pine stem segments at different distances from the inoculation site. No significant differences were observed in the number of *BxICD1* dsRNA, *eGFP* dsRNA, and the non-dsRNA solution-treated nematodes at pine stem segments 1-2 and 2-3 cm below the inoculation site. However, the number of *BxICD1* dsRNA-treated nematodes was significantly lower compared with *eGFP* dsRNA and non-dsRNA solution-treated nematodes isolated from pine stem segments 3-4 cm below the inoculation site. No nematodes were isolated from pine stem segments 4-5 cm below the inoculation point in all treatments (**Figures 6E, F**).

In addition, *BxICD1* dsRNA, *eGFP* dsRNA, and the non-dsRNA solution-treated nematodes were cultured on a PDA medium covered with *Pestalotiopsis* sp. After eight days, no significant differences were observed in the amount of nematodes and fungi mycelium in different treatments (**Supplementary Figure 3**). The above results showed that *BxICD1* affected *B. xylophilus* parasitism and migration in *P. massoniana*, but did not affect the feeding rate and reproduction of *B. xylophilus* in fungi.

## 4 Discussion

Effectors are the key factors affecting PPNs virulence and parasitism against hosts (Goverse and Smant, 2014). In recent years, a growing number of PPN candidate effectors have been identified by transcriptome sequencing. More than 100 candidate effectors from *B. xylophilus* infecting *P. thunbergii* have been obtained, while a few have been functionally characterized (Tsai et al., 2016; Hu et al., 2019). Interestingly, it was reported that different proteins were detected in the secretomes of *B. xylophilus* during the infection of different pine hosts (Silva et al., 2021). Accordingly, we screened effectors from *B. xylophilus* infecting *P. massoniana* for the first time. As result, 394 candidate effectors were identified through transcriptome data and bioinformatics analysis. Among them, 210 were different from the candidate effectors of *B. xylophilus* infesting *P. thunbergii* and were not annotated in the HMMER homology database, which might be pioneers effectors against *P. massoniana*. One of them, named BxICD1, was found to be significantly upregulated in the early parasitic stages of *B. xylophilus* and specifically present in the esophageal gland cell, a classical effector secretion organ of nematodes (Mitchum et al., 2013). Furthermore, the signal peptide of BxICD1 was confirmed to be functional based on yeast system assays. These data suggest that BxICD1 is a novel effector of *B. xylophilus*.

In this study, we found that BxICD1 could induce PCD in the extracellular space of *N. benthamiana*, which was dependent on NbBAK1. As is known, PCD has different effects on different nutritional pathogens with different lifestyles and feeding strategies. Necrotrophic pathogens need to kill cells and tissues of host plants, and then absorb nutrients. Therefore, PCD facilitates the parasitism of necrotrophic pathogens (Laluk and Mengiste, 2010). However, biotrophic pathogens must acquire nutrients directly from living plant cells and tissues without immediately killing host cells or tissues. Thus, PCD is often detrimental to biotrophic pathogens (Gebrie, 2016). Additionally, hemibiotrophs are biotrophic in the early stage and necrotrophic in the late stage of parasitism. Therefore, PCD inhibits the infection by hemibiotrophs in the early parasitism stage, while it promotes hemibiotroph colonization and facilitates pathogen transition from a biotrophic to a necrotrophic state in the late parasitism stage (Jia et al., 2000; Orbach et al., 2000; Qutob et al., 2002). PPNs are biotrophic pathogens, and it is generally considered that PCD negatively affects nematode parasitism. For example, the potato protein Gpa2 recognizes the *Globodera pallida* effector Gp-Rbp-1 and triggers PCD, which inhibits nematode parasitism (Sacco et al., 2009). A hypersensitive response-like reaction was observed in the *Meloidogyne graminicola*-resistant rice plants but not in the *M. graminicola*-susceptible rice plants under nematode infection (Cabasan et al., 2014). To survive, PPNs secrete effectors to suppress PCD. In recent years, many PPN effectors with PCD-inhibiting properties have been identified, especially from sedentary endoparasitic phytonematodes, e.g., root-knot nematodes and cyst nematodes (Postma et al., 2012; Chen et al., 2017; Song et al., 2021; Chen et al., 2022). In the migratory endoparasitic phytonematode *B. xylophilus*, six effectors were also found to inhibit PCD (Hu et al., 2021; Wen et al., 2021, Wen et al., 2022; Zhang et al., 2022; Hu et al., 2022a; Qiu et al., 2023). Interestingly, five effectors from *B. xylophilus* were confirmed to have the ability to activate PCD. One of them not only induced PCD but also inhibited nematode parasitism. However, BxSapB1, BxSapB2, and BxSapB3, induced PCD but at the same time also enhanced nematode virulence (Huang et al., 2019; Hu et al., 2019, Hu et al., 2020; Zhao et al., 2020; Shinya et al., 2021). In the present study, we found that BxICD1 also promots parasitism of *B. xylophilus* based on RNAi gene silencing assays. This seemingly paradoxical phenomenon was also observed in effectors from oomycetes and fungi, such as PsXEG1 (Ma et al., 2015) from *Phytophthora sojae*, Avh238 (Yang et al., 2017) from *P. essential*, PlAvh142 (Situ et al., 2020) from *Peronophythora litchi* and Fg12 (Yang et al., 2020) from *F. graminearum*. Two hypotheses have been proposed to explain this phenomenon. First, the accumulation of effectors is insufficient to induce PCD under natural conditions. Second, other interacting effectors or proteins could suppress PCD activated by effectors during the successful parasitism of pathogens (Ma et al., 2015; Yang et al., 2017; Situ et al., 2020).

This study demonstrated that BxICD1 contributed to nematode migration in *P. massoniana*, but was not involved and did not affect nematode feeding and reproduction in fungi based on the RNAi assays results. It has been reported that pine trees develop resistance by inducing pine resin secretion and lignification in the resin canals. Lignin biosynthesis in cell walls can effectively inhibits the migration of *B. xylophilus* (Kusumoto et al., 2014). In pine trees, highly virulent *B. xylophilus* strains can rapidly destroy the resin-secreting parenchyma cells of cortical and xylem resin canals before cell wall lignification (Jin and Ye, 2007), while weakly virulent strains do not have such capacity. Taken together, the effector BxICD1 has the capacity to induce PCD, resulting in the destruction of parenchyma cells in resin canals, which is beneficial for *B. xylophilus* virulence and migration.

## Data availability statement

The datasets presented in this study can be found in online repositories. The names of the repository/repositories and accession number(s) can be found below: BioProject, PRJNA1027064.

## Ethics statement

The manuscript presents research on animals that do not require ethical approval for their study.

## Author contributions

ZL: Writing – original draft, Writing – review & editing, Data curation, Formal analysis, Investigation, Methodology, Software. HW: Writing – original draft, Methodology, Writing – review & editing, Formal analysis, Investigation. YC: Writing – original draft, Formal analysis, Investigation. XS: Writing – original draft, Formal analysis, Investigation. XH: Writing – original draft, Formal analysis, Investigation. QH: Investigation, Writing – original draft, Formal analysis. KZ: Methodology, Writing – review & editing, Writing – original draft. JL: Writing – original draft, Writing – review & editing, Methodology. BL: Writing – review & editing, Formal analysis, Investigation, Methodology.

## Supplementary material

Supplementary Figure 1 Localization of *BxICD2* mRNA in the intestine terminus of *Bursaphelenchus xylophilus* by *in-situ* hybridization. DIG, digoxygenin; Scale bars = 50 µm.

Supplementary Figure 2 Multiple sequence alignment and phylogenetic analyses of BxICD1 (BXY_0304900). **(A)** Multiple sequence alignment of the BxICD1 protein with other homologous sequences from *Bursaphelenchus xylophilus*. **(B)** The phylogenetic tree of BxICD1 protein homologs.

Supplementary Figure 3 BxICD1 does not affect reproduction or fungal feeding rate in *Bursaphelenchus xylophilus* **(A)** The propagation rate of *B. xylophilus* after culture on *Pestalotiopsis* spp. for 8 days. Data represent the means, and the error bars represent ± standard deviation of six biological replicates. **(B)** The fungal feeding rate of *B. xylophilus* grown on *Pestalotiopsis* spp. Two independent experiments were performed with similar results. dseGFP, dsBxICD1 and CK indicate nematodes inoculated in *eGFP*-, *BxICD1-* and non-dsRNA solution, respectively.
