# Supplementary figures and images for: A Bursaphelenchus xylophilus effector BxICD1 inducing plant cell death, concurrently contributes to nematode virulence and migration
Source: Front Plant Sci. 2024 Feb 28;15:1357141. doi: 10.3389/fpls.2024.1357141 (PMC10933036; doi:10.3389/fpls.2024.1357141)

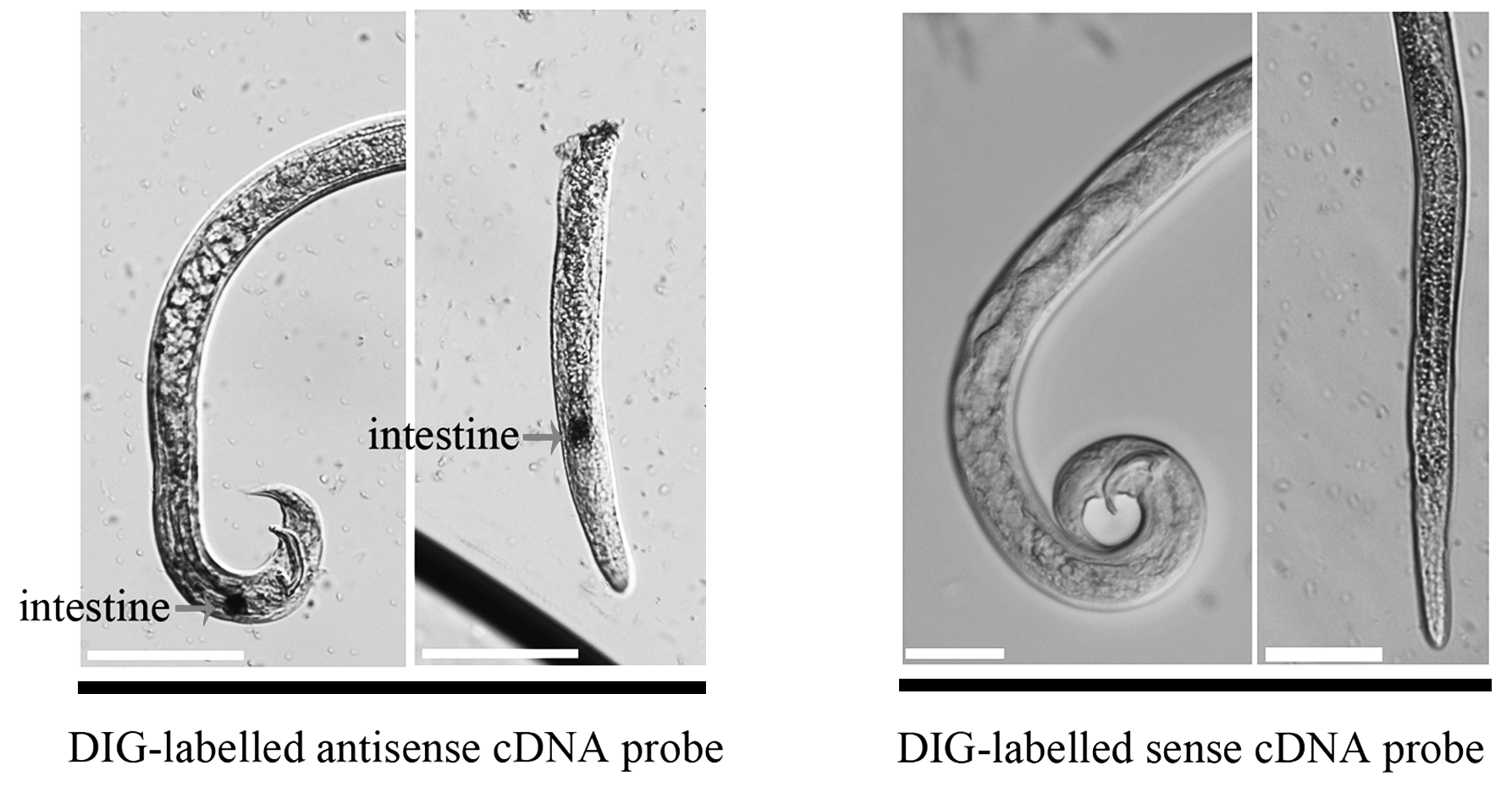

Supplement: Supplementary Figure 1 — Localization of BxICD2 mRNA in the intestine terminus of Bursaphelenchus xylophilus by in-situ hybridization. DIG, digoxygenin; Scale bars = 50 µm. [file Image_1.tif]

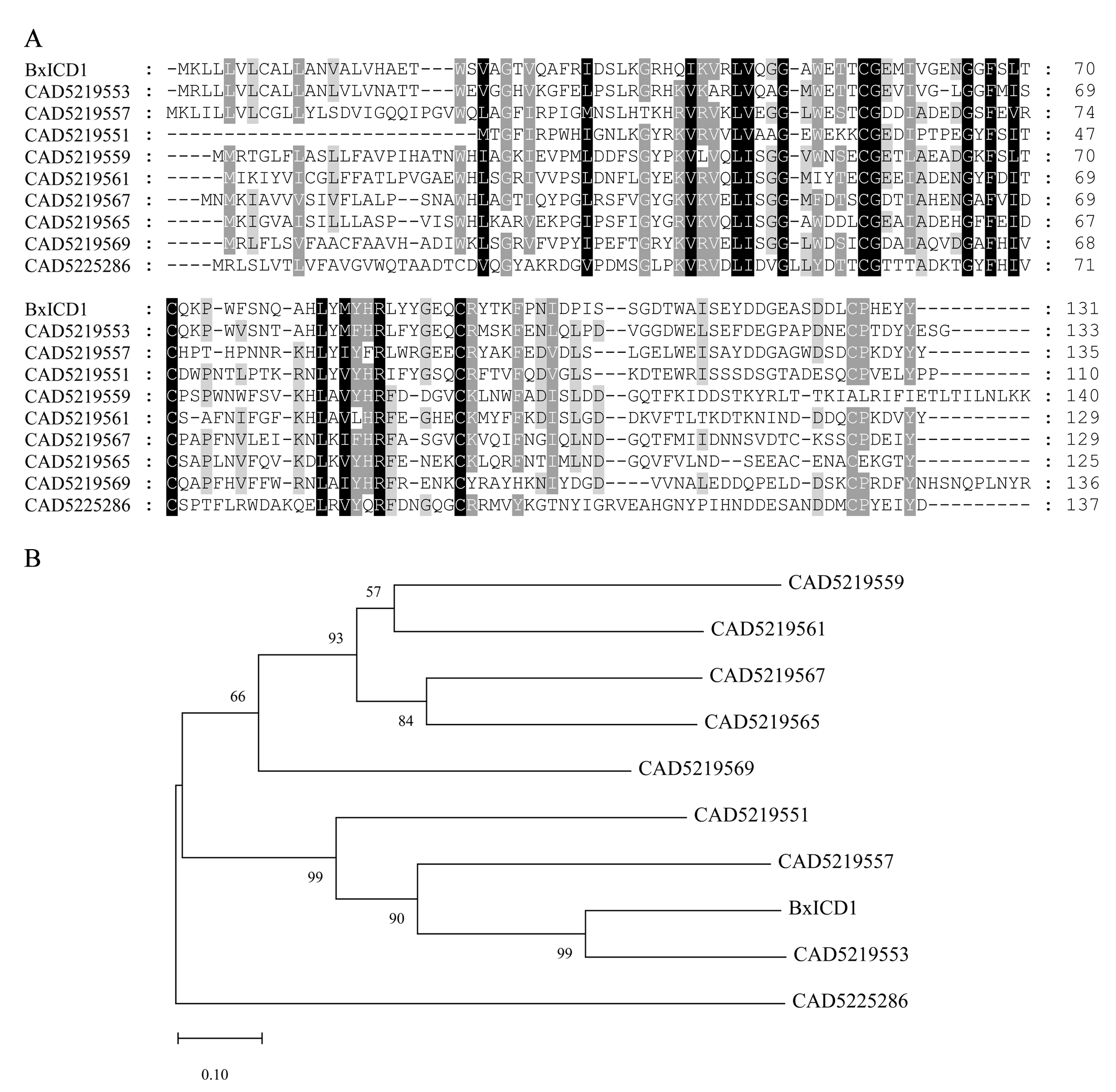

Supplement: Supplementary Figure 2 — Multiple sequence alignment and phylogenetic analyses of BxICD1 (BXY_0304900). (A) Multiple sequence alignment of the BxICD1 protein with other homologous sequences from Bursaphelenchus xylophilus. (B) The phylogenetic tree of BxICD1 protein homologs. [file Image_2.tif]

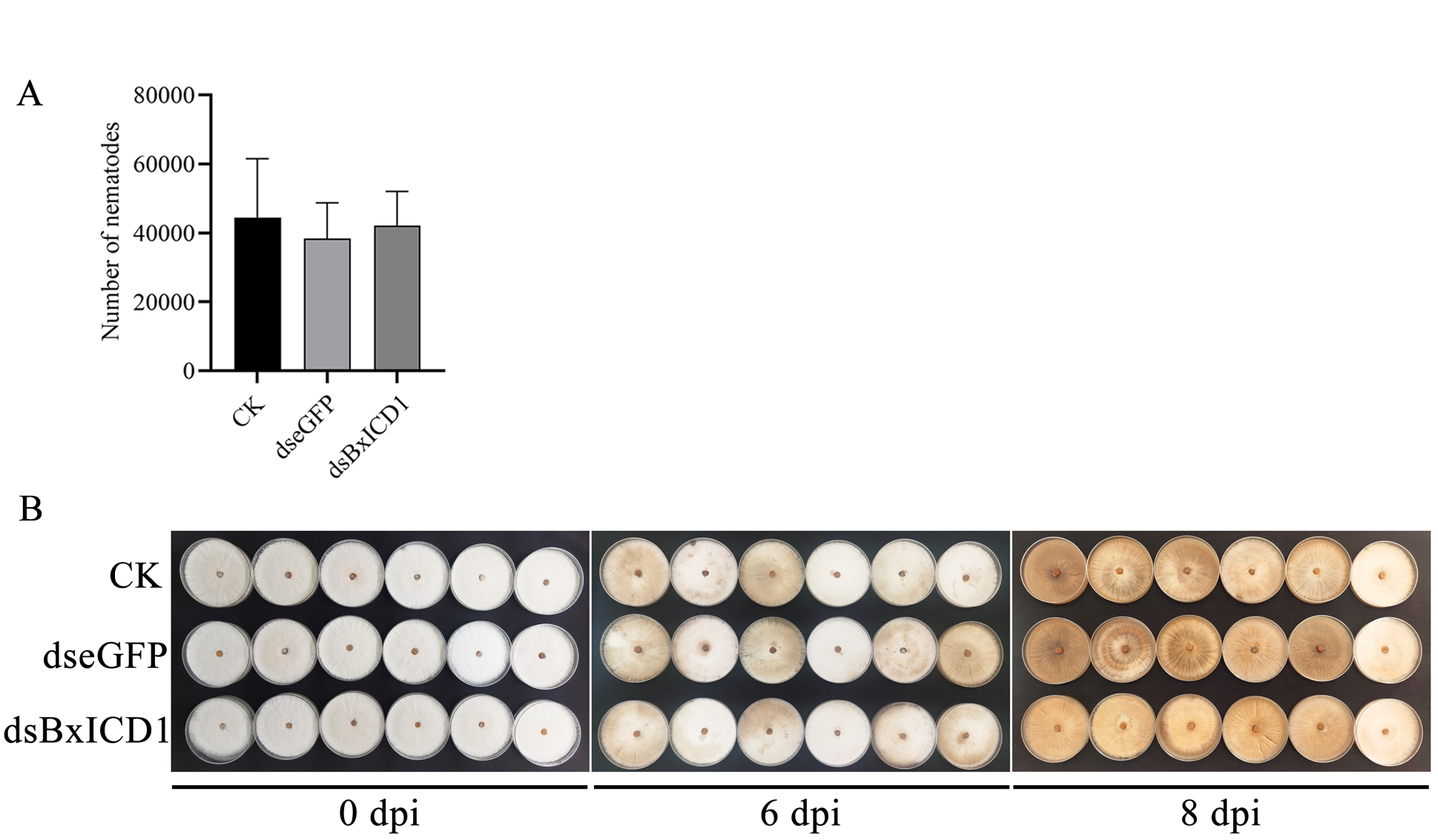

Supplement: Supplementary Figure 3 — BxICD1 does not affect reproduction or fungal feeding rate in Bursaphelenchus xylophilus (A) The propagation rate of B. xylophilus after culture on Pestalotiopsis spp. for 8 days. Data represent the means, and the error bars represent ± standard deviation of six biological replicates. (B) The fungal feeding rate of B. xylophilus grown on Pestalotiopsis spp. Two independent experiments were performed with similar results. dseGFP, dsBxICD1 and CK indicate nematodes inoculated in eGFP-, BxICD1- and non-dsRNA solution, respectively. [file Image_3.tif]
